# Nutritional Interventions in Older Persons with Type 2 Diabetes and Frailty: A Scoping Systematic Review

**DOI:** 10.3390/jcdd11090289

**Published:** 2024-09-18

**Authors:** German C. Giraldo Gonzalez, Luz M. González Robledo, Isabel C. Jaimes Montaña, Angela M. Benjumea Salgado, Sayda M. Pico Fonseca, Martha J. Arismendi Solano, Claudia L. Valencia Rico

**Affiliations:** 1 Doctorado en Ciencias de la Salud, Facultad de Ciencias de la Salud, Universidad de Caldas, Manizales 170004, Colombia; angela.benjumea@ucaldas.edu.co (A.M.B.S.); isabel.jaimes@ucaldas.edu.co (I.C.J.M.); sayda.39420135777@ucaldas.edu.co (S.M.P.F.); martha.arismendi@castillo.edu.co (M.J.A.S.); cvalencia@ucm.edu.co (C.L.V.R.); 2 Facultad de Medicina, Universidad Autónoma del Estado de Morelos, Cuernavaca 62350, Mexico; luz.gonzalez@uaem.mx; 3 Departamento de Salud Pública, Universidad de Caldas, Manizales 170004, Colombia; 4 Departamento Clínico, Universidad de Caldas , Manizales 170004, Colombia; 5 Departamento de Salud Pública y Epidemiología, Pontificia Universidad Javeriana de Cali, Cali 760031, Colombia; 6 Centro de Investigación y Atención en Salud del Magdalena Medio-CISMAG, Barrancabermeja 687031, Colombia; 7 Programa de Enfermería, Grupo de Investigación en Enfermería, Universidad Católica de Manizales, Manizales 170001, Colombia

**Keywords:** frailty, type 2 diabetes mellitus, older people, nutritional intervention

## Abstract

In the elderly, the coexistence of type 2 diabetes mellitus (T2DM) and frailty is frequent. Much has been described about pharmacological management and glycemic control goals. However, there is a knowledge gap in terms of the objectives and characteristics of interventions, especially nutritional ones, for this population. A scoping review was performed to document the objectives, characteristics, and results of nutritional interventions in older people with T2DM and frailty. The five-stage framework of Arksey and O’Malley was used, as was the PRISMA extension for scoping reviews. The results stand out for three trends, as follows: (1) experimental studies with multicomponent intervention physical exercise programs and nutritional programs based on educational processes or behavioral intervention; (2) observational studies with an association of the kind of diet assessed by scales and their relation to stages of frailty; (3) a review that updates recommendations on pharmacological and non-pharmacological, diet, exercise, management, as well as glucose control goals for diabetes in frail older persons. Finally, the evidence shows that management of T2DM in older adults with frailty requires goals and interventions tailored to their functional capacity and health condition. The exercise, diet, and education programs reviewed have demonstrated their effectiveness in improving physical performance, reducing the risk of frailty or progression to more advanced stages, and achieving better glycemic control.

## 1. Introduction

A demographic characteristic of today’s society is the continual increase in the number of older adults. Indeed, along with aging, we find a greater prevalence of chronic non-communicable diseases such as type 2 diabetes mellitus (T2DM) [1], which can decrease life expectancy by up to 6 years [2], increase the probability of hospitalization, degrade quality of life, and lead to a high rate of cardiovascular complications [3,4].

Aging is also accompanied by the appearance of frailty, which may be present in 32–48% of older adults with diabetes and can predispose the older person to be highly vulnerable and have less resistance to stressors or complications [5,6,7,8,9,10,11].

The likelihood of complications affecting quality of life (neuropathy, retinopathy, chronic kidney disease) or that are potentially fatal (cardiovascular disease) stemming from T2DM increases where there is worse control of the disease [12,13]; therefore, maintaining adequate diabetes control should always be included among the clinical objectives. Although hyperglycemia is not the only disorder generating complications, it does double the risk of being hospitalized and increases mortality when significant reductions in glucose levels are sought [1,14,15].

To establish good diabetes control and as a pillar of treatment, it is necessary to provide nutritional recommendations that are essential to avoiding hyperglycemic or hypoglycemic spikes. Therefore, in older adults, clinical management guidelines recommend providing optimal nutrition and adequate protein intake, except in those patients who present obesity [16,17,18]. However, nutritional intake decreases with aging: some people present anorexia, there are food access problems, the quality of nutrients ingested is low, and because they are diabetic, they tend to have quite a few restrictions on calories and fats. This nutritional deficit is related to frailty [6,19,20,21,22].

Recognizing the best nutritional recommendations for a frail, diabetic older adult is essential to reaching specific elder-care objectives, which include not only metabolic monitoring but also frailty monitoring. Although there is a high prevalence of older people with diabetes among people with frailty [23], existing studies have focused on the effects of nutritional intervention on functional capacity [24,25,26].

The results commonly found in the available literature imply that although the combination of nutritional support, cognitive training, and physical activity has a greater impact [27], nutrition alone can also significantly improve the frail state of frail and prefrail elderly people [25,27]. However, there is a knowledge gap in terms of the objectives and characteristics of interventions, especially nutritional ones, for the population in question. Details are missing from their description regarding the type of diet, modality, duration, accompaniment, and scenarios, among others. It is necessary to analyze and compile the results of prior studies that serve as a tool for health professionals to identify nutritional intervention options to have better results that can affect the quality of life of older people with T2DM and frailty.

This research explores the scope of nutritional interventions in elderly people with type 2 diabetes and frailty, aiming to identify knowledge gaps for future research that could support evidence-based treatment decisions and delay the onset of frailty.

## 2. Materials and Methods

Based on a protocol previously developed by the authors (which is available on request), a systematic scoping review was conducted using the methodological framework proposed by Arksey and O’Malley, which consists of five steps: (1) identification of the research questions; (2) identification of the relevant literature; (3) article selection; (4) data extraction; and (5) summary and report on the results [28].

This detailed protocol ensured that the review was rigorous and provided a solid foundation for exploring nutritional interventions aimed at this vulnerable population, effectively identifying knowledge gaps, and suggesting future directions for research and clinical practice. Additionally, the PRISMA extension for scoping reviews (PRISMA-ScR) was used to complement the methodological framework, contributing to the transparency and reproducibility of the review process. The protocol can be found in the Supplementary File.

This kind of review, based on a systematic focus to map the evidence on a topic [29] (in this case, nutritional interventions in older adults with T2DM and frailty), enables the use of different approaches to explore the evidence published on the question, identify characteristics or factors related to the phenomenon under study, and provide compiled and summarized evidence to increase knowledge on the topic, support decision-making in patient management, innovate on ways to research the topic, and/or identify knowledge gaps [30,31,32].

Scoping reviews are particularly useful in areas where there are few randomized controlled trials to conduct systematic reviews since they address questions beyond those related to the effectiveness of the intervention and the generalization of results [33]. Furthermore, they are characterized by addressing broad topics to which many different study designs may be applied. The research questions are less specific than in other types of systematic reviews, and therefore, it is not necessary to assess the quality of the included studies [28].

The methodological framework within which the current review is based was supplemented with the PRISMA extension for scoping reviews (PRISMA-ScR) [29].

### 2.1. Eligibility Criteria

Included articles were derived from original research published in peer-reviewed academic journals, had neither geographic nor methodological restrictions, were published between 1 January 2018 and 15 October 2022 in English, Spanish, and/or Portuguese, and described nutritional and/or diet-type interventions in the study population (older adults with T2DM and frailty). References outside the established timeframe that did not specify the nutritional and/or diet aspects or outcome variables were excluded. The inclusion of articles in English, Spanish, and Portuguese in the review is justified due to their extensive coverage in research on diabetes and nutrition and because they are the predominant languages in key areas such as Latin America and the Iberian Peninsula. This selection reflects the capabilities of the research team, allowing for accurate analysis without the need for translations that could introduce errors. Limiting the languages also maintains the quality and consistency of the review, optimizing the management of the literature.

### 2.2. Information Sources and Search

Four databases were consulted: PubMed, Web of Science, Scopus, and Science Direct. The search strategy was based on different combinations of controlled vocabulary with the terms Mesh, DeCS, and keywords: frailty, diabetes mellitus, nutritional intervention, diet, older adults, older persons, older people, older patients, older individuals, persons 65 years and older, and older population, using Boolean operators. Filters associated with the selection criteria, such as temporality, language (English, Spanish, and Portuguese), and availability of full text, were applied. Management guidelines, reflection articles, editorials, and gray literature were excluded.

### 2.3. Selection of Sources of Evidence and Data Charting Process

Once the references obtained in the first search were organized in a database built in the Microsoft Excel statistics package (365 Microsoft subscription, https://www.microsoft.com/en-us/microsoft-365/excel, accessed on 5 June 2022) and duplicates were eliminated, a team of six health professional reviewers with experience in internal medicine (one cardiologist), geriatrics (one doctor and one nurse), and public health (one doctor, one epidemiologist, and one nutritionist) independently reviewed each of the references obtained, first by title and then by abstract. Based on this review, studies were chosen as potentially relevant for the review. At a later stage, they were reviewed in detail based on the full text. Disagreements were discussed and resolved within the review team.

### 2.4. Literature Research Results

**Figure 1:**
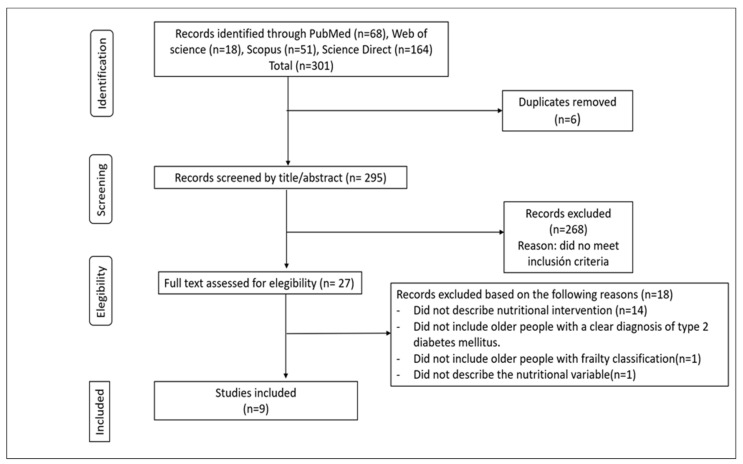
PRISMA Flowchart Diagram.

The initial search produced 301 titles, and after eliminating duplicates, the number of references was 295. A preliminary assessment of the document based on title and abstract was conducted, and 27 were selected as directly related to the research questions. Finally, after an exhaustive review of the texts, nine articles were selected that met the inclusion criteria (see study selection flowchart in Figure 1).

## 3. Results

### 3.1. Characteristics of the Studies Included in the Review

Of the nine studies selected, most (*n* = 4) were conducted in European countries, mainly Spain, followed by the United States (*n* = 3) and Japan (*n* = 2). All the articles analyzed were written in English (Table 1).

On discrimination by design and study type, three tendencies stand out among the manuscripts found. First, investigations derived from experimental studies with multicomponent intervention (physical exercise programs and nutritional programs based on educational processes or behavioral intervention) [34,36,37,38,39]. Second, observational studies have shown an association between the kind of diet assessed by scales and their relation to stages of frailty [39,40,41]. Third, one review article that updates recommendations on pharmacological and non-pharmacological (diet, exercise) management, as well as glucose control goals for diabetes in frail older persons [42] (Table 2).

### 3.2. Outcome Variables

The outcome variables of the studies reviewed are diverse and correspond not only to nutritional aspects but are also targeted to changes obtained in functional conditions, diabetes control goals, diet-related frailty, and cost-effectiveness indicators for the interventions, as described below.

- Functional changes based on nutritional and multicomponent interventions.

The Short Performance Physical Battery (SPPB) was the main instrument used to assess functional changes, reporting short- and long-term benefits in three of the studies reviewed. In the first, by Rodríguez-Mañas et al. in 2019 [38], they demonstrated that the intervened group presented better averages on the SPPB, with an increase of 0.85 on scores (95% CI, 0.44–1.26, *p*-value < 0.001) than the control group, with significant differences in the three components, especially that of getting up from a chair five times. The second study, headed by Izquierdo in 2021 [36], shows an improvement in mobility performance with an average of 36.1% in week 18 (*p* < 0.001) and of 10.2% in week 68 (*p* < 0.05) and a significant improvement in the balance component (95% CI [−0.15, −0.85], *p* = 0.008). Likewise, it reports that after 2 years of follow-up, the SPPB values observed continued to be significantly higher than baseline, and in general, the intervention enabled a reduction in the condition of frailty in participants (21 people in frail condition and 25 in prefrail at baseline versus 8 in frail condition and 9 in prefrail at study end). In the third study by Jiwani et al. in 2022 [34], the SPPB sub-scores were reported separately, showing significant improvement solely in balance tests (95% CI [−0.15, −0.85], *p* = 0.008).

On the other hand, the study by Izquierdo et al. [37] used other functional markers in addition to SPPB, such as 1RM (estimated maximum dynamic force of one repetition) that showed improvements between 45.2% and 57.2% in week 18 (*p* < 0.01–0.001) and muscle power, in which significant improvements were observed after weeks 10 and 18 (*p* < 0.01–0.001, SD (0.58–0.60). After one year of follow-up, 1RM was significantly lower than that observed in week 18, but the tendency was significantly greater than that observed at the beginning of the study, with no meaningful changes after the second intervention period.

The study by the Look AHEAD Research Group in 2022 [35], in which they compared an intensive lifestyle intervention to a control group as functional change, reported regression between stages of frailty reflected in weakness, slow speed, low physical activity, and tiredness over the study period and showed a significant difference when comparing the average total frailty score at the beginning of 1.61 (SD ± 1.15) with the final study score of 0.94 (±0.94), for a difference between averages of 0.67 (95% CI [1.15, 0.18], *p* = 0.01).

- T2DM control goals

Some interventions, like the one by Jiwani in 2022, show an improvement in glycosylated hemoglobin (HbA1c) of 3% with no significant differences [34], and the study by Izquierdo et al. in 2021 [36] showed a significant decrease in HbA1c after the first intervention of 16 weeks (*p* < 0.05, SD (0.16); however, there were no additional differences between the other time points (i.e., after weeks 52, 68, and 104). On the other hand, in their study, Nishimura et al. reported in 2019 a reduction of 2.3% in fasting blood glucose [40].

In terms of anthropometric measures, the intervention by Jiwani et al. [34] reported an average weight loss of 7.3 lbs. (95% CI, 3.92–10.73), with males responding with greater loss (−7.9 lbs., *p* < 0.05). Another parameter that reported significant changes in this study was the body mass index (BMI), with an average of 32.5 kg/m^2^ at the end of the intervention (95% CI [1.69, 0.61]).

- Frailty associated with diet

The study by Hayakawa et al. of 2021 [39] sought the relationship between the results of the applied dietary variety questionnaire in older persons with T2DM and the presence of frailty. In the logistic regression analysis, the group of diabetics with limited dietary variety reports an Odds Ratio (OR) of frailty of 5.03 (95% CI, 2.05–12.35). Poorly varied diet and T2DM were independently associated with frailty. The study by López-García et al. in 2018 [41] assessed the association of the Mediterranean diet with low frailty risk in older women with T2DM and reported that the increase in consuming the Mediterranean diet was associated with a 28% reduction in the risk of frailty (95% CI, 19–36%). This was especially observed in diets reported to have high fruit and vegetable content.

On the other hand, the Sanz-Cánovas et al. literature review of 2022 [42] found that frailty is a related but independent entity in the evolution of diabetes; high levels of cytokines like TNFα, IL-1, IL-5, or IL-6 that are present in diabetic patients induce insulin resistance and mitochondrial dysfunction, which alter lipid peroxidation, generate lipid increase in muscle cells, and favor the development of frailty and sarcopenia. Regarding HbA1c goals in the elderly with T2DM and frailty, it is recommended that they be tailored and propose an HbA1c goal of 7.5–8.5%, emphasizing the maintenance of low blood glucose variability. The nutritional intervention is a diet of 30 kcal/kg of body weight/day, with a protein quantity of 1.0 to 1.2 g/kg of body weight per day to maintain and restore muscle mass in those over 65 years old, maintain normal vitamin D levels, and highly recommended the Mediterranean diet. Physical exercise was recommended in different modalities and adapted to the functional conditions (including severe physical disability) and health of each person with T2DM.

- Cost-effectiveness

The cost-effectiveness analysis showed that the participants included in one of the multicomponent intervention studies reviewed (MID FRAIL Study) [36,37] on average incurred lower health care costs per patient as compared to the usual care of 428.02 EUR (2016) per patient per year. This is particularly due to lower hospitalization costs [540.93 EUR (2110.08)] in the intervention group compared to the usual care group [11.76.75 EUR (3730.92)], which represents a savings of 630.82 euros (*p* = 0.041). This multicomponent intervention is associated with an average cost of 332 EUR per patient in the follow-up year, with average annual health care costs 25% higher among patients receiving usual care.

## 4. Discussion

The management of elderly diabetic patients with frailty is a topic of growing interest and study. The literature analyzed reports multicomponent intervention studies where education and behavioral therapy are the dietary strategies described, which, in conjunction with the physical exercise protocol, show greater effectiveness in reducing the risk of frailty, blood glucose levels, and their variability, with a favorable impact on physical performance. The observational studies relate a highly varied diet and a Mediterranean-style diet to significant decreases in the risk of frailty [39,41].

The multicomponent intervention studies emphasize exercise protocols and their results based on functional indicators. Three studies used the SPPB mobility parameters; one of them shows significant improvement in three components (balance, gait speed, and getting up from a chair five times) [38], while the other two are only in the balance component [34,36]. This may be explained by the difference in protocols and their characteristics (type of program, intensity, frequency, and duration). The Rodríguez-Mañas et al. study from 2019 [38], in which improvement in three of the SPPB components is shown, was the only one that used a control group and that states a supervised exercise program. The dietary intervention was based on education and behavioral therapy, based on educational sessions that emphasized knowledge of the disease and behavior change regarding caring for and controlling T2DM, with key health messages such as maintaining an optimal nutritional status, avoiding both hypo- and hyperglycemia, and setting goals and receiving feedback. A diet style with different food groups, their portions, and frequency are not specifically described. The favorable results in frailty and blood glucose control are based on the combined effect of the intervention; there is no sub-analysis of the impact of dietary intervention in older adults with diabetes and frailty. This has two perspectives. One is multicomponent management, which includes exercise, education, and social participation and has greater effectiveness than isolated management based on overall goals. The other is one where these intervention studies describe clear and complete protocols for physical activity, though not for the dietary intervention, which opens a panorama of aspects yet to be resolved.

One way to explain the effectiveness of these programs is supported by the pathophysiological basis of T2DM. Frailty is a frequent clinical syndrome in people with this condition, and it is the greatest determining factor for disability in diabetic people. Frailty is found in 32–48% of adults 65 years and older with diabetes [43] and is associated with adverse events and reduced survival [44]. T2DM and frailty share pathophysiological mechanisms, such as chronic hyperglycemia and changes in insulin levels that lead to alterations in the metabolism of carbohydrates, fats, and proteins, which in turn lead to insulin resistance and a deficit of insulin secretion with protein loss in muscles [45]. Glycemic control and lower variability in blood glucose based on adherence to pharmacological and non-pharmacological management (adherence to dietary recommendations, control of body weight, motivation, education, etc.), together with a physical exercise plan that is also multicomponent (those that include in the protocol different modalities such as aerobic, strength, power, and endurance exercises) [43], explains and justifies the efficacy reported in these intervention studies.

Few studies have been conducted on the role of nutrition in the increased risk of frailty among older people with T2DM. The Hayakawa et al. study from 2021 [39] found that a poorly varied diet is an independent factor related to frailty (Table 2); a varied diet refers to consuming different types of foods at least once a week. Diabetes and management recommendations targeted at the global population place the older adult group at risk of eating a poorly varied, limited diet and at risk of malnutrition, sarcopenia, and frailty. Diversifying food intake can help older people consume enough energy and nutrients without disturbing blood glucose control. The Lopez-García et al. study from 2018 [41] found an association between the Mediterranean-style diet and a reduction in frailty risk. The definition of a Mediterranean-style diet is diverse. In general, it contains low levels of saturated fats and high levels of vegetable oils. In the daily quantity of foods and nutrients, this is represented as three to nine portions of vegetables, one to two portions of fruit, one to thirteen portions of grains, and eight portions of olive oil, which contains 18% monounsaturated fat, 9% saturated fat, and 33 g of fiber per day [46].

A diet rich in plants and vegetables, characterized by a high level of minerals and flavonoids with anti-inflammatory and antioxidant properties, helps to restore homeostasis, which results in muscle maintenance even in old age [47,48]. This is an explanation that can be attributed to the decrease in frailty risk, which is associated with greater oxidative stress and a lower level of circulating antioxidants [49]. This reduction in risk has also been observed in other studies; the one by Brunner et al. from 2018 [50] showed that those who eat fruits and vegetables less than once a day in the 45–55 age range showed a significantly greater risk of developing frailty 18 years later as compared to those who ate them more than once a day (adjusted OR = 1.29, 95% CI = 1.05–1.58).

Older adults present changes in body composition that range from loss of height, muscle tissue, and bone density to an increase in and redistribution of adipose tissue, which leads to changes in weight and, thus, in body mass index. Food intake is frequently underestimated by older people, who also have a pro-inflammatory state and chronic diseases, facilitating caloric protein depletion; therefore, the diet should be sufficient, complete, and balanced [51,52]. The review of the literature included in this scoping review [42] compiles guidelines from different scientific societies and expert recommendations, and the section on diet and nutrition gives specific recommendations regarding daily energy intake, protein intake for maintaining and restoring muscle mass, vitamin D, and a Mediterranean-style diet (Table 2). It emphasizes tailored management for this population, which is an important, timely approach to caring for this group of people. It also invites determining dietary patterns (size, quality, and frequency) based on intervention studies that can be extrapolated, recognizing this as a current gap in knowledge.

## 5. Conclusions

Management of T2DM in older adults with frailty requires goals and interventions tailored to their functional capacity and health condition. The exercise, diet, and education programs reviewed have demonstrated their effectiveness in improving physical performance, reducing the risk of frailty or progression to more advanced stages, and achieving better glycemic control.

### Strengths and Limitations

There are several strengths of this scoping review. It is a search of updated literature that makes clear the kinds of studies conducted and the main results that are applicable and able to be extrapolated to the population of interest. It gives an account of nine studies published on this specific topic and orients the need to continue studying the relationship between diet and nutrition in the control of older adult patients with T2DM and frailty, as well as their effects and results. Only articles published in three languages (English, Spanish, and Portuguese) were eligible because of the ability to interpret the content by the researchers.

As a limitation, the lack of homogeneity regarding the recommendations or interventions given to frail, diabetic older adults is recognized. Other aspects are variability in sample sizes, follow-up time, and the training program established, which could affect results. Including only articles in English, Spanish, and Portuguese also represents a limitation in the review, as it potentially excludes valuable studies published in other languages. This restriction could miss relevant research conducted in other regions of the world where these languages are not predominant, limiting the generalizability of the results and potentially biasing the overall understanding of the topic under investigation. Thus, while it facilitates the review process, this choice could affect the breadth and diversity of the evidence incorporated into the analysis.

## Figures and Tables

**Table 1:** Characteristics of the studies included in the review.

Primary Author, Year	Country	Study Type
Jiwani, 2022 [34]	USA	Experimental
Look AHEAD Research Group, 2022 [35]	USA	
Izquierdo, 2021 [36]	Spain	
Peña, 2021 [37]	7 European countries (Belgium, Czech Republic, United Kingdom, France, Germany, Italy, and Spain)	
Rodríguez, 2019 [38]		
Hayakawa, 2021 [39]	Japan	Observational
Nishimura, 2019 [40]	Japan	
López, 2018 [41]	USA	
Sanz, 2022 [42]	Spain	Review

Source: own elaboration.

**Table 2:** Objective, population/scenario, and protocol or recommended measurement of the selected studies.

Study	Objective	Population/Scenario	Intervention or Recommended Measurement
Jiwani R, et al. (2022) [34]	To determine the feasibility of a behavioral lifestyle intervention with mobile health technology for self-monitoring of diet and physical activity to improve frailty in overweight/obese older adults.	Twenty community-dwelling overweight/obese older persons: self-reported T2DM diagnosis and frailty identified through five criteria (weight loss, exhaustion, low energy expenditure, grip strength, and gait). University of Texas Health Science Center at San Antonio (UTHSCSA)	Behavioral intervention based on a lifestyle program enhanced with mobile health technology. - Ten behavioral therapy sessions for six months, with an in-person or virtual modality. Frequency: weekly in month 1, biweekly in months 2 and 3, and monthly in months 4 to 6. Each session lasted between 60 and 90 min. - Missed sessions were substituted with an individual recovery session or a phone consult. - Content of the behavioral sessions: self-monitoring, goal setting, feedback, mindful eating, talking back negative thoughts, social support, problem-solving, social relapse, problem-solving, and relapse prevention, among others. - Introduction of a weight loss goal of 5–7% based on calorie and fat intake based on their current weight and a physical activity goal gradually increasing to 175 min/week. - Adaptation of the Fitbit activity wristband for self-monitoring of diet and physical activity. Downloading the application to participants’ smartphones. Providing information on how to record food (portion size, calories, and fats) and physical activity (duration and kind of activity) using the Fitbit wearable activity tracking application.
Look AHEAD Research Group (2022) [35]	To compare the prevalence of the frailty phenotype among intervention groups in long-term follow-up	5145 people with T2DM, body mass index (BMI) ≥ 25 kg/m^2^ (≥27 kg/m^2^ if using insulin) and diabetes T2DM	Intensive Lifestyle Intervention (ILI): Individual and group sessions were taught intensively for 1 year with weekly visits during the first 6 months, 3 times per month during the following 6 months, and less intensively with monthly visits from years 2 to 4. During years 2 to 4, individual appointments were held at least once per month, and additional contact was made by phone, mail, or e-mail. From year 5 on, at least 2 visits were conducted per year, as well as annual campaigns to promote adherence. The personalized intervention for weight loss included self-monitoring of dietary intake together with behavioral strategies for portion control and other eating behaviors. Education on increasing physical activity and instruction of at least 175 min of moderately intensive physical activity were conducted per week. Group sessions on topics related to physical activity covered methods for exercising safely and the benefits of exercise. Diabetes Support and Education (DSE): This involved group classes on diet, exercise, and social support offered 3 times per year during years 1 to 4, which decreased to annual group classes thereafter.
Izquierdo, et al. (2021) [36]	To analyze the effects of a program composed of resistance training and nutritional interventions on functional capacity, maximal strength, and muscle power in frail persons with type 2 diabetes.	52 participants with T2DM, frail or prefrail as identified by Fried’s phenotype. “MIDPOW” sub-study was conducted in 5 geriatric services in public hospitals in Spain.	Nutritional and educational program: - Taught at each clinical intervention center. Included a nutritional evaluation and 7 separate educational sessions of 45 min each offered by a researcher or nutritional therapist twice a week for 3.5 to 4.0 weeks. - The educational sessions included 4 to 8 participants. - Topics: behavior change and health messages, such as maintaining an optimal nutritional status and avoiding hypoglycemia. - Use of two education support manuals called “Improving nutritional status and knowledge of diabetes” (manual for trainers and participants). Exercise program: - 2 Weeks of evaluation, pre-training, and familiarization with the exercises. - 16 weeks of resistance exercises. Intensity of 2 to 3 series of 8 to 10 repetitions, with a load equivalent to 40% to 80% of the maximum estimated for one repetition (1RM). Frequency of 2 sessions per week.
Peña-Longobardo, et al. (2021) [37]	To estimate the incremental cost-utility ratio (ICUR) of a multi-modal intervention in frail and pre-frail patients aged ≥ 70 years with T2DM compared to the usual care group focused on quality-adjusted life years (QALY) in European countries.	Older people with T2DM for at least 2 years and with frailty criteria. Countries: Belgium, Czech Republic, Italy, Germany, France, United Kingdom and Spain. Economic evaluation of the MID FRAIL Study	Multicomponent intervention - Physical exercise program: Frequency: twice a week. Supervised resistance training for 16 weeks (from weeks 2 to 18) using two specific machines that offered leg extension and leg press exercises. - Nutritional education program: Seven separate 45-min sessions moderated by a doctor in small groups of four to eight subjects. The education program aimed to increase knowledge and understanding of diabetes, develop practical skills for diabetes self-monitoring, and improve and ensure safe blood glucose control. The comparator was the usual care group, which was specified as having no additional active intervention and a population of hospitalized older persons with T2DM.
Rodríguez-Mañas, et al. (2019) [36]	To evaluate the efficacy of the multi-modal intervention in the functional performance of older persons over 70 years of age with T2DM and pre-frailty or frailty stage. MID FRAIL Study	Over 70 years old, with T2DM, and classified as pre-frail or frail. In the hospital and at primary care sites in 7 European countries (Belgium, Czech Republic, United Kingdom, France, Germany, Italy and Spain)	Intensive control group: - Exercise program against resistance: 2-week pre-training and learning phase, followed by a 16-week program. Two 45-min exercise sessions per week. The strength exercises were adapted for individual functional ability using variable resistance training machines (Exercycle SL, BH Group, Vitoria, Spain), two to three series of 8 to 10 repetitions with a load equivalent to 40–80% 1RM. Two muscle exercises for the lower limbs (leg press and bilateral knee extension) were included. - Nutritional education program: Seven educational sessions taught by a trained researcher, 45-min sessions, twice per week for 3.5 to 4 weeks, on the same day as the exercise intervention, in the study’s initial phase. The educational sessions were given only to a small number of subjects (four to eight) and were focused on behavior change and key health messages, such as maintaining an optimal nutritional status, avoiding hypoglycemia, and caring for diabetes symptoms. Each nutritional therapist or researcher was provided with two internally developed manuals: “Improving Nutritional Status and Diabetes Knowledge” (Trainers’ Manual) and “Improving Nutritional Status and Diabetes Knowledge” (Participant Guide) to support the educational sessions. Usual care group: Defined as the level of routine care that an older participant with T2DM would expect to receive from their health care system, including their primary care physician.
Hayakawa, et al. (2021) [39]	To establish the relationship between a poorly varied diet in elderly patients with diabetes and the presence of frailty. Takashimadaira Study and Otassha Study	≥70 years old, 2 years of follow-up 2016–2018 annual health checkup since 2011, ages between 65 and 84 years Assessment of diabetes mellitus: Self-reporting and database review. Assessment of frailty: Performed by trained researchers, the J-CHS Scale was derived from operationalizing the Fried phenotype. Difference reference points for grip strength H: 28 kg/force and M < 18 kg/force and gait speed under 1 mt/sec.	Assessment of varied diet score: Dietary variety (DVS) questionnaire includes 10 food categories: meat, seafood, eggs, soy products, milk, green and yellow vegetables, seaweed, potatoes, fruit, and fats and oils. For each category, a score of 1 is allocated if the participants’ answers are “eats almost every day” and a score of 0 for all the remaining answers. The total score ranges from 0 to 10. Scores of 0 to 4 are defined as high variety (low scores), and scores > 5 are low variety (high scores).
Akiko Nishimura, et al. (2019) [40]	Describe the sex-related differences in diabetes-specific factors underlying the development of frailty in older persons with T2DM.	Older persons, 60 to 80 years old, with T2DM and no limitation in basic care activities (ADL) Frailty. Frailty was evaluated using the Kihon Checklist (KCL).	Demographic characteristics: Age, sex, academic background, family structure, work status, irregular lifestyle (irregular bedtimes or irregular eating habits), drinking habits, and smoking habits were obtained from medical records and a general questionnaire. Body compositions: Bioelectric impedance methods. Diabetes-related factors: Treatment of diabetes, complications, and blood test results were obtained from medical records. Hypoglycemia was confirmed by reviewing the self-monitored blood glucose levels or hypoglycemia episodes during the last 3 months. Hypoglycemia was defined as a blood glucose level of ≤70 mg/dL. Diabetes self-management performance was measured using the Summary of Diabetes Self-Care Activities (SDSCA) measure. The higher the subscale mean score, the higher the level of self-care practice.
López García, et al. (2018) [41]	To assess whether a Mediterranean diet is associated with a lower risk of frailty among diabetic women.	Women > 60 years old with T2DM Prospective cohort study Nurses’ Health Study (NHS) Frailty assessment: FRAIL scale (fatigue, low resistance, low aerobic capacity, having several Illnesses, and loss of weight during the previous year). Self-reported scale and data adapted from previous surveys.	Dietary assessment: Food frequency questionnaires (FFQ) were distributed to participants in 1990, 1994, 1998, 2000, 2006, and 2010. Adherence to a Mediterranean diet was derived from information obtained via the FFQ using alternative scoring. For the Mediterranean diet score (aMED), the cumulative average of the scores of all available questionnaires from the T2DM diagnosis through the end of follow-up was used. Physical activity: Reported as the average time spent per week during the previous year in specific activities (outdoor walking, jogging, and cycling). The time spent on each activity was multiplied by the typical energy expenditure, expressed in metabolic equivalents, and then added for all activities.
Sanz-Cánovas J. (2022) [42]	To review the therapeutic aspects of T2DM in elderly patients with frailty and/or sarcopenia.	People over 75 years old with T2DM and frailty.	Sarcopenia and frailty in diabetic people: Frailty is proposed as a related but independent entity in the evolution of diabetes. High levels of cytokines like TNFα, IL-1, IL-5, or IL-6, present in a diabetic patient, induce insulin resistance and mitochondrial dysfunction that alter lipid peroxidation, increasing lipids in muscle cells, which favors the development of frailty and sarcopenia. Glucose monitoring goals in frailty: Glucose monitoring goals for patients of advanced age must be adapted to their functional status, cognitive state, co-morbidities, and life expectancy. It is important to maintain low glycemic variability, not just protect them from hyper- or hypoglycemia. HbA1c of 7.5–8.5%, fasting glucose levels for light to moderate frailty 90–100 mg/dL and 150–180 mg/dL for severe frailty. Non-pharmacological management: Exercise: Resistance and aerobic exercises prevent and treat the decrease in strength and muscle mass in the elderly. Resistance training positively affects the neuromuscular system and increases hormone concentrations and the rate of protein synthesis. Multiple types of physical exercise have been studied. Warm-up of 5 min and 3 min of cool-down. Depending on the patient’s initial physical state, low-intensity walking is recommended, and if not possible, riding a stationary bike. Other exercises can include squats, knee bends, hip extensions, or flexion. For patients with a severe physical disability, exercises such as knee extension, ankle circles, arm lifting, chair pushing (tricep extension), a tennis ball can be squeezed, the neck turned while sitting, or making leg circles. Diet: A plan tailored according to nutritional status, level of physical activity, illness status, and tolerance in general recommends an energy intake of 30 kcal/kg of body weight/day for aging. A protein intake of 1.0 to 1.2 g/kg of body weight per day is needed to maintain and restore muscle mass and function in patients over 65 years old. Maintain normal levels of vitamin D. The Mediterranean diet is highly recommended.

Source: own elaboration.

## Data Availability

All data used in this study are available by request from the corresponding authors.

## Supplementary Materials

The following supporting information can be downloaded at: https://www.mdpi.com/2308-3425/11/9/289/s1. The protocol with which the revision was built can be found in the Supplementary File.
