# Supplementary material for: Nutritional Interventions in Older Persons with Type 2 Diabetes and Frailty: A Scoping Systematic Review
Source: J Cardiovasc Dev Dis. 2024 Sep 18;11(9):289. doi: 10.3390/jcdd11090289 (PMC11605221; doi:10.3390/jcdd11090289)
Supplement: Supplementary file 1 [file jcdd-11-00289-s001.zip › jcdd-2984743-supplementary.pdf]

Research Seminar IV

Doctorate in Health Sciences

Caldas University, Colombia, South America, 2024

Systematic Literature Review Protocol

Topic: nutritional intervention in older people with type 2 Diabetes Mellitus and frailty

Introduction

Older people with type 2 Diabetes Mellitus (T2DM) tend to experience accelerated aging, which leads to a higher risk of developing frailty at a younger age (1). T2DM, sarcopenia and frailty are mutually associated and frequently coexist. These 3 entities have common pathophysiological mechanisms such as insulin resistance, chronic inflammation and mitochondrial dysfunction; their presence is a marker of worse prognosis in older people (2). The prevalence of frailty syndrome is higher in adults with DM2 than in the general population (3).

Aim

To document the objectives, characteristics and results of nutritional interventions in older people with T2DM and frailty.

Methodology

It is proposed to carry out a scoping review using the methodological framework proposed by Arksey and O'Malley (2005) (1), which consists of five steps: 1) identification of the research questions; 2) identification of relevant literature; 3) selection of articles; 4) data extraction and 5) summary and reporting of the results complemented with the PRISMA extension for this type of review (PRISMA-ScR) (2).

PICO᾽S questions:

What is the effect of a nutritional intervention on the clinical outcomes of frail older adults with type 2 diabetes mellitus?

P: frail elderly over 65 years of age with type 2 Diabetes Mellitus.

I: nutritional intervention

C: without nutritional intervention

O: clinical outcomes

Article sources and search strategy:

It is proposed to identify electronic resources from various sources. Four databases will be consulted: PubMed, Web of Science, Scopus and Science Direct.

Keywords for search strategy

Frailty AND diabetes mellitus AND “intervention”

"Nutrition intervention AND diabetes IN elderly with frailty"

"Diabetes IN frailty AND nutrition intervention" "Glycemic control with nutritional intervention IN frail diabetic patient"

"Frailty AND diabetes AND nutrition intervention"

As an example, for the Science Direct database, the detailed search was performed as follows: ("Frail Elderly" [MeSH Terms] OR ("Frail Elderly" [All Fields] and "Diabetes Mellitus" [All fields]) and " nutrition intervention "[All fields]).

Pubmed search

Search engines:

"Nutrition intervention AND diabetes IN elderly with frailty", "Diabetes IN frailty AND nutrition intervention", "Frailty AND diabetes AND nutrition intervention"

Total 68 references: selected for second analysis: 13 references.

Selection criteria

Search engines:

**Inclusion criteria**	**Exclusion criteria**	**Elimination Criteria**
Articles published in the last 5 years.<br><br>Articles in English, Spanish and/or Portuguese.<br><br>Publications derived from research by any approach and design.<br><br>Articles describing nutrition interventions.	Management guides.<br><br>Thought pieces.<br><br>Editorials.<br><br>Gray literatura.<br><br>Intervention articles that do not explain the outcome variable.	No access to full text

Articles that classify frailty by phenotypic and frailty index.<br><br>Definition of glycemic control by HbA1c values.		

Workplan:

The review will be carried out by a team of 7 reviewers, made up of 6 PhD students in Health Sciences at the University of Caldas, and a teacher who is an expert in systematic reviews.

Phase 1: Planning: corresponds to this protocol.

Phase 2: Review:

The steps necessary to identify relevant electronic resources for the systematic review are search, filter, eligibility and inclusion.

The records will be thoroughly screened to eliminate non-relevant records by reading the titles, abstracts and finally the full texts, according to the established selection criteria.

Phase 3: Report:

The bibliographic findings of the literature were reported by each researcher in an Excel table, they were qualified according to eligibility criteria by two experts and the number of articles to be included was defined according to inclusion criteria.

Bibliography

1. Arksey H, O'Malley L. Scoping studies: towards a methodological framework. Int J Soc Res Methodol [Internet]. 2005;8(1):19–32. Available from: doi:

https://doi.org/10.1080/1364557032000119616

2. Tricco AC, Lillie E, Zarin W, O'Brien KK, Colquhoun H, Levac D, et al. PRISMA Extension for Scoping Reviews (PRISMA-ScR): Checklist and Explanation. Ann Intern Med [Internet]. 2018;169(7):467–73. Available from: doi: 10.7326/M18-0850

3. Collard RM, Boter H, Schoevers RA, Oude Voshaar RC. Prevalence of frailty in community-dwelling older persons: a systematic review. J Am Geriatr Soc 2012;60(8):1487-92
